# Heat intolerance: the challenge of returning to duty post-exertional heatstroke

**DOI:** 10.3389/fspor.2026.1860120

**Published:** 2026-06-03

**Authors:** Jingchun Song

**Affiliations:** 1 Intensive Care Unit, The 908th Hospital of Chinese PLA Logistic Support Force, Nanchang, China; 2 Nanchang Key Laboratory of Thrombosis and Hemostasis, Nanchang, China

**Keywords:** diagnostic criteria, exertional heatstroke, heat intolerance, heat tolerance assessment, heat tolerance rehabilitation, return to duty

## Abstract

Exertional heatstroke, a critical and severe condition precipitated by intense physical exertion in high-temperature settings, frequently culminates in a protracted state of heat intolerance among athletes and military personnel. This condition not only jeopardizes individual health but also impedes their capacity to resume their duties. Emerging research indicates that the chronic heat intolerance that follows exertional heatstroke is markedly distinct from the pathophysiological trajectories of acute heat injury, encompassing intricate alterations across various systems and levels. The present paper provides a comprehensive narrative review of the underlying mechanisms of heat intolerance post-exertional heatstroke, along with the diagnostic criteria and therapeutic strategies for heat endurance rehabilitation. Furthermore, it scrutinizes the shortcomings inherent in current diagnostic and therapeutic methodologies and anticipates future research trajectories, with the ultimate goal of enhancing the return-to-duty efficiency of individuals who have suffered from heatstroke.

## Key points

1. A comprehensive description is provided to elucidate the pathophysiological mechanisms underlying heat intolerance following exertional heatstroke.
2. It outlines the specific criteria for diagnosing heat intolerance using heat tolerance tests, along with key points for quality control and safety during implementation, as well as the limitations of such tests.
3. The paper comprehensively examines the principal therapeutic approaches for treating heat intolerance, encompassing heat acclimation, physical therapy, and cognitive therapy.
4. A thorough summary is presented regarding the optimal timing for initiating heat acclimation training, the selection of training modalities, and the duration of treatment courses.

## 1 Introduction

Exertional heat illness is an acute condition precipitated by intense physical exertion in high-temperature settings, with varying degrees of severity ranging from heat cramps to heat exhaustion and exertional heatstroke. The latter is the most severe form, characterized by a life-threatening multi-organ dysfunction syndrome resulting from severe thermoregulatory failure (1). It has been reported that the incidence rates of heat exhaustion and exertional heatstroke among U.S. military personnel are 172.7 and 31.7 per 100,000 person-years, respectively, with a 4.4% recurrence rate for individuals with a history of exertional heatstroke (2). Current research suggests that recurrent heatstroke is primarily associated with a persistent state of heat intolerance (3). Unlike the pathophysiology of acute heat injury, this persistent state of heat intolerance involves complex mechanisms such as aberrant heat shock protein expression, immune dysregulation, and impaired tissue repair, the specifics of which remain to be fully elucidated (3). Furthermore, the heat tolerance test (HTT), currently used as a decision-making tool for the return to duty of patients with exertional heatstroke, still exhibits significant deficiencies in accuracy and universality (4). Therefore, this paper will introduce three aspects: the mechanisms underlying the persistent state of heat intolerance following exertional heatstroke, the current status of heat endurance recovery techniques worldwide, and future research priorities.

## 2 Mechanisms of heat intolerance following exertional heatstroke

During heat stress, conditions of high temperature and humidity can result in a disparity where the body's heat production surpasses its capacity for heat dissipation, leading to heat accumulation. To counteract this, cutaneous vasodilation is markedly increased, and profuse sweating is employed as a cooling mechanism, which, in turn, can diminish the effective circulating blood volume. Concurrently engaging in intense physical exertion can further aggravate this volume deficit due to the congestion of striated muscles, culminating in a critical imbalance between heat generation and dissipation, thereby precipitating exertional heatstroke. A diagnosis of exertional heatstroke is warranted when an individual's core body temperature soars above 40℃, accompanied by a constellation of multi-organ dysfunctions with central nervous system impairment as the predominant feature (1). Individuals who have suffered from exertional heatstroke may develop persistent heat intolerance, which manifests as a reduced ability to adapt to thermal stress, characterized by a more protracted and insidious pathological progression. Those with heat intolerance are prone to an excessive and rapid rise in core temperature beyond safe thresholds during standard heat tolerance assessments, coupled with attenuated sweating responses and other manifestations of autonomic dysregulation. The discourse among scholars worldwide regarding heat intolerance oscillates between viewing it as an inherent human trait versus a reactive state, with the prevailing consensus attributing heat intolerance to a confluence of genetic predispositions and the capacity for heat acclimatization (3). The following section primarily describes the pathophysiological and molecular mechanisms underlying persistent heat intolerance.

### 2.1 Chronic thermoregulatory dysfunction

Heatstroke causes extensive damage to the thermoregulatory system. The acute thermal injury during the initial phase lays the pathological groundwork for chronic heat intolerance. A sharp rise in core temperature above 40℃ can directly damage the hypothalamic thermoregulatory center, potentially altering the temperature set point or blunting the central response to heat stress, thereby establishing the neurological basis for chronic thermoregulatory dysfunction (1). Systemic pathological processes may further impair heat dissipation. Heatstroke can trigger Systemic Inflammatory Response Syndrome (SIRS), which damages vascular endothelial cells, leading to degradation of the endothelial glycocalyx, impaired vasomotor regulation, and disruption of intercellular junction proteins. These microcirculatory changes reduce skin blood flow and sweating, thereby decreasing peripheral heat dissipation (5). Myocardial injury is another key factor. Heat stress can directly harm cardiomyocytes, causing cardiac dysfunction and reduced cardiac output reserve. In hot environments, the body must balance increased skin blood flow for heat dissipation with the demands of active muscles. An impaired heart may fail to deliver adequate cardiac output, exacerbating the rise in core temperature. Individuals with compromised cardiovascular function have a limited ability to increase stroke volume, cardiac output, and skin blood flow, which significantly raises their risk of heatstroke (6).

### 2.2 Abnormal regulation of heat shock proteins

**Figure 1:**
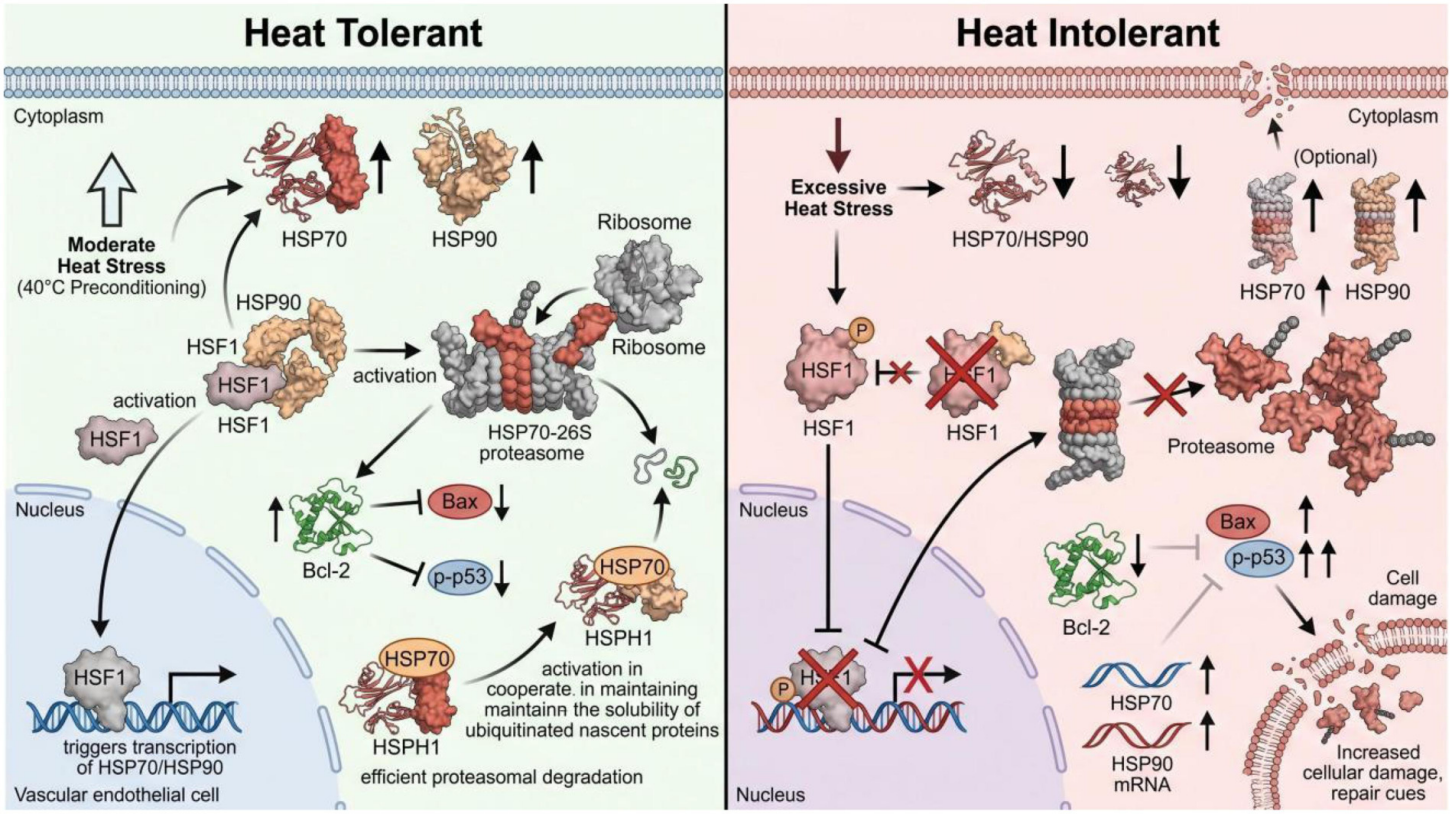
The mechanism of heat shock proteins in heat stress. HSF, heat shock transcription factor; HSP, heat shock protein.

Cellular stress responses constitute a pivotal molecular defense against environmental stressors and viral infections. Upon exposure to heat stress, cells activate a suite of mechanisms—including the heat shock protein (HSP) response, unfolded protein response, mitochondrial stress signaling, and DNA damage response—to restore cellular homeostasis (6). Within this arsenal, HSPs function as molecular chaperones, central to maintaining cellular integrity and shielding cells from thermal injury. The HSP family, encompassing HSP100, HSP90, HSP70, HSP60, and small HSPs, aids in heat stress management through protein folding, clearance of misfolded proteins, apoptosis regulation, and signal transduction (7). Moderate heat stress can substantially boost cellular heat tolerance and facilitate tissue repair by upregulating HSP70 and HSP90 expression. Studies demonstrate that brief heat preconditioning (e.g., 40℃ exposure) can enhance HSP70 and the anti-apoptotic Bcl-2 protein, while curbing the activity of pro-apoptotic factors p-p53 and Bax, thus improving vascular endothelial cell survival during subsequent heat stress (8) (Figure 1). HSP70 promotes the association of the 26S proteasome with translating ribosomes, accelerating the degradation of misfolded proteins and restoring protein synthesis (9). HSP70 and its co-chaperone HSPH1 act together during the acquired thermotolerance phase to maintain the solubility of ubiquitinated nascent proteins, ensuring their efficient degradation by the proteasome.

Heat intolerance is associated with dysregulated expression of HSPs such as HSP70 and HSP90. Excessive heat stress suppresses HSP expression and impairs cellular clearance of misfolded proteins, thereby exacerbating cell damage and delaying recovery of heat tolerance. Furthermore, aberrant activity of heat shock transcription factor 1 (HSF1) can lead to dysregulated HSP expression. HSF1 is normally sequestered in an inactive state by HSP90 but is released upon heat stress to initiate HSP transcription (9). Abnormal phosphorylation of HSF1 or disruption of the HSP90-HSF1 interaction directly affects the expression of HSP70 and HSP90, aggravating cellular damage (10). Individuals with heat intolerance exhibit persistently elevated mRNA levels of HSP70 and HSP90 during heat tolerance testing (HTT), yet show only a minimal corresponding increase in HSP70 protein levels (11). This pattern is linked to a sustained lack of HSF1 mRNA responsiveness during heat stress in heat-intolerant individuals.

### 2.3 Abnormal immune response

Heat stress can elicit a spectrum of immune-inflammatory responses, contingent upon its intensity. Moderate-Intensity Continuous Training (MICT), characterized by 30–60 min of exercise at 50%–70% of VO2max, is known to bolster anti-inflammatory cytokines such as IL-10, suppress pro-inflammatory cytokines like Tumor Necrosis Factor-alpha (TNF*α*), mitigate oxidative stress, and enhance the expression of intestinal tight junction proteins ZO-1 and occludin. Additionally, MICT can elevate the abundance of beneficial gut bacteria, including Lactobacillus and Bifidobacterium, thereby fortifying intestinal barrier integrity and microbial diversity (12). In contrast, High-Intensity Interval Training (HIIT), which involves exercise at >70% maximum oxygen consumption (VO_2_max) for durations exceeding 90 min, may surpass the physiological thresholds of trainees. This can lead to blood redistribution to active muscles and peripheral vasculature, potentially inducing gastrointestinal ischemia (13). Such ischemia may disrupt intestinal permeability through the “gut-immune axis,” precipitating endotoxemia and heightened inflammatory responses during exercise. This is evidenced by elevated levels of inflammatory mediators (e.g., C-reactive protein and IL-6), increased gut colonization by Escherichia coli and Clostridium, and compromised intestinal barrier function.

**Figure 2:**
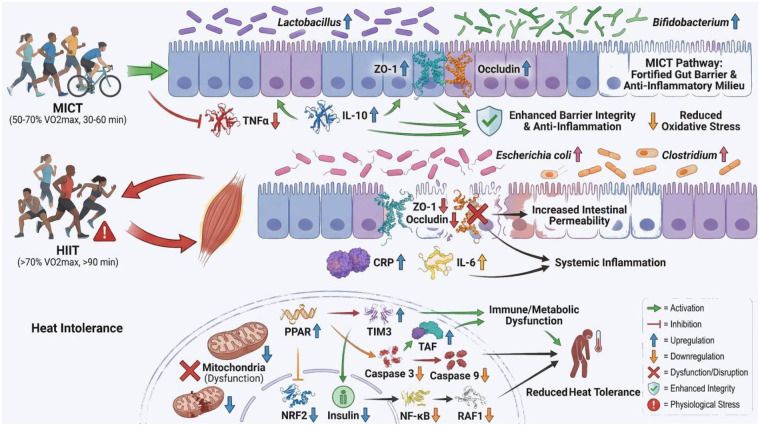
Immune responses under varying intensities of heat stress. HIIT, High-intensity interval training; MICT, moderate-intensity continuous training; VO_2_max, maximum oxygen consumption.

Current research indicates that persistent heat intolerance is linked to an altered immune profile post-heat stress. An Israeli study categorized 40 individuals with a history of exertional heatstroke into heat-intolerant and heat-tolerant cohorts based on HTT outcomes and subsequently analyzed their peripheral blood mononuclear cells (PBMCs) for gene expression changes (14). The study revealed that, in comparison to the heat-tolerant group, the heat-intolerant group exhibited significant gene expression alterations in PBMCs. These changes were predominantly associated with mitochondrial dysfunction, downregulation of exercise endurance-related factors such as NRF2 and insulin, and upregulation of peroxisome proliferator-activated receptor (PPAR) expression. Notably, there were substantial increases in the expression of key differential genes TIM3 and TAF, while the expression of Caspase 3, Caspase 9, NF-*κ*B, and RAF1 was significantly reduced (Figure 2).

### 2.4 Incomplete recovery of organ damage

Exertional heatstroke is a critical condition that can precipitate significant organ damage, with the potential to result in fatal multi-organ dysfunction. This condition often necessitates an extended convalescence. A retrospective analysis of 2,529 cases of exertional heatstroke revealed that biomarkers indicative of acute kidney injury, such as blood urea nitrogen, creatinine, and urinalysis, typically reach their peak on the day of onset and normalize within 24 to 48 h. Similarly, indicators of muscle and liver damage usually peak within four days of the onset and can remain elevated for 2 to 16 days (15). Currently, the recovery of heatstroke patients is assessed based on the absence of subjective symptoms and the normalization of routine laboratory tests. However, recent clinical observations have indicated that some patients, who were asymptomatic and had normal plasma creatine kinase levels three months post-treatment and discharge, still exhibited significant rhabdomyolysis on magnetic resonance imaging. Other patients presented with signs of myocardial cell edema and fibrosis (16, 17). This suggests that the complete recovery of organ damage in patients with exertional heatstroke may require a longer period of time at the anatomical level. Such cellular-level organ damage may be one of the mechanisms underlying the persistent state of heat intolerance.

### 2.5 Genetic factors

The susceptibility to exertional heatstroke is influenced not only by environmental conditions and individual physiological traits but also significantly by genetic predispositions. Recent human studies have employed whole-exome sequencing to delve into the genetic underpinnings of exertional heatstroke. A comprehensive genetic analysis of 64 British military personnel with a history of exertional heatstroke revealed 51 non-polymorphic, potentially pathogenic variants. Notably, the variants RYR1 p., PYGM p., and CACNA1S p. were identified as being associated with exertional heatstroke, suggesting their involvement in the disruption of skeletal muscle calcium homeostasis, oxidative metabolism, and membrane excitability (18).

In another study, 15 soldiers who had experienced exertional heatstroke were found to harbor two missense variants in the TRPV1 gene, which plays a pivotal role in thermoregulation and nociception. *In vitro* functional assays demonstrated that these genetic variants markedly altered the TRPV1 channel's response to its natural agonist, capsaicin, thereby validating TRPV1 as a novel candidate gene implicated in exertional heatstroke (19).

Furthermore, in Australia, a single nucleotide polymorphism (SNP) analysis of heat shock protein (HSP) genes was conducted on 48 military individuals with a history of exertional heatstroke. The findings indicated a correlation between genetic variations at the g.31829044 locus of the HSPA1B gene and heat tolerance, underscoring the role of genetic factors in the body's response to heat stress (20).

## 3 Diagnosis of heat intolerance

The determination of whether individuals who have suffered from exertional heatstroke can safely return to training or other activities post-recovery is a critical clinical decision. In this context, the HTT serves as an objective physiological assessment tool, pivotal in ascertaining the presence of ongoing heat intolerance and thereby informing the decision to return to activity (21). The essence of the HTT is to simulate conditions of heat stress and to evaluate an individual's capacity to adapt to heat by closely monitoring key physiological parameters, such as core body temperature and heart rate (22). However, the timing of initiating the test, the specific procedural details, and the threshold criteria for diagnosing heat intolerance are all factors that significantly impact the accuracy and safety of the assessment.

### 3.1 Diagnostic criteria for heat intolerance

**Figure 3:**
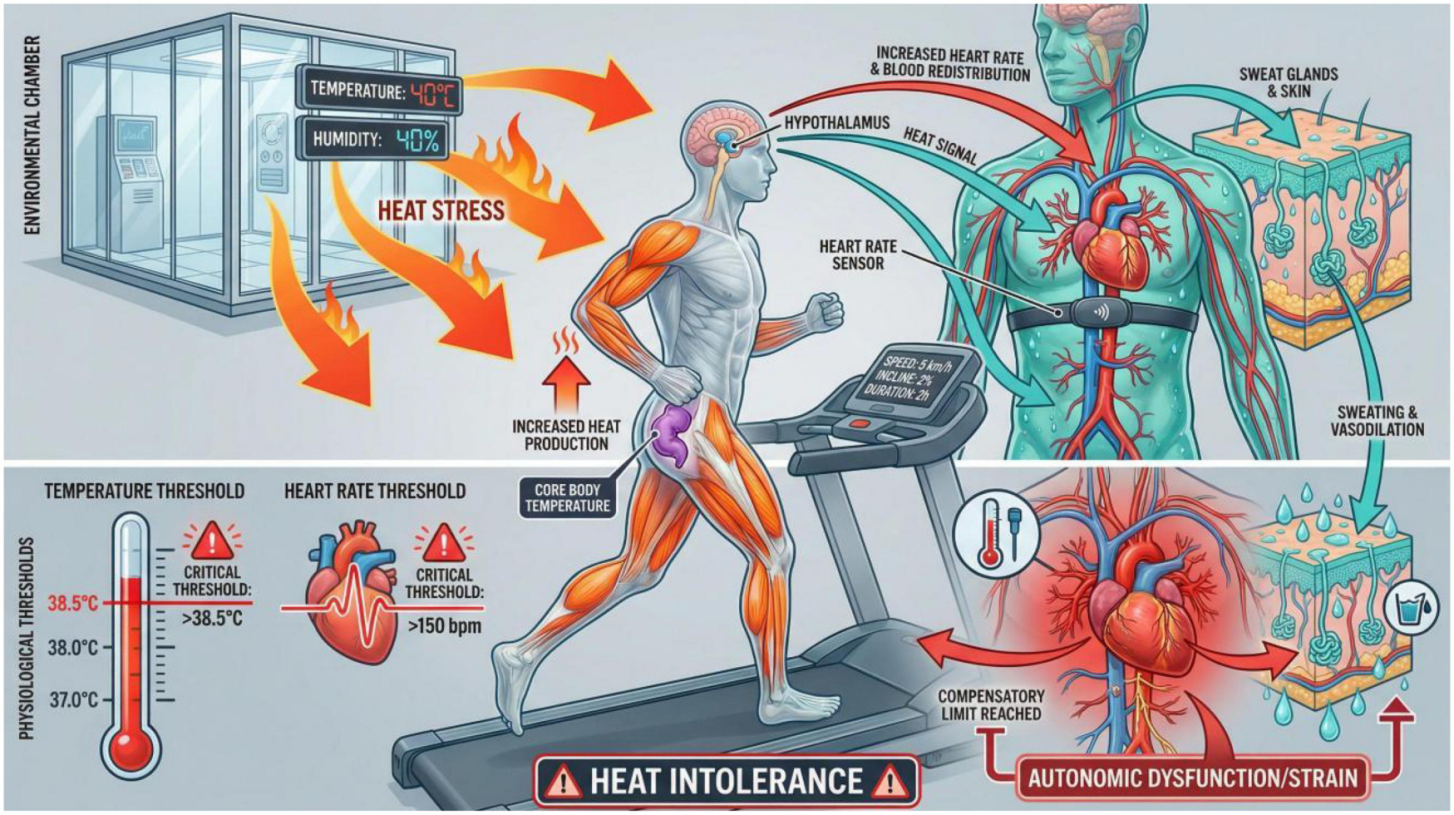
Testing and diagnosis of heat intolerance. For core temperature monitoring, rectal temperature is the first recommended method, with a value exceeding 38.5°C considered positive.

The diagnostic criteria for heat intolerance were initially established by Israeli Defense Force (IDF). This protocol is customarily executed within a meticulously controlled laboratory setting designed to replicate extreme heat stress conditions. A standardized test environment is pivotal for ensuring the comparability and reliability of the results, typically established in an artificial climate chamber with a temperature of 40℃ and a relative humidity of 40% (23). This hot and humid milieu is intended to maximally elicit the thermoregulatory responses of the subjects, thereby accurately gauging their heat tolerance status. Throughout the test, subjects are tasked with performing continuous exercise on a treadmill. The quintessential exercise regimen involves subjects walking at a pace of 5 km/h, often with a 2% incline, to maintain a consistent, moderate-intensity exercise load for a duration of 2 h (24, 25) (Figure 3). This exercise intensity approximates 50% of the individual's VO_2_max, aiming to generate sufficient endogenous heat to challenge the thermoregulatory system.

In the HTT, core body temperature and heart rate thresholds are the principal objective criteria for ascertaining heat intolerance. Should a subject's rectal temperature surpass 38.5℃ or their heart rate exceed 150 beats per minute during the test, they are deemed “heat intolerant” (22). Research has demonstrated that when core temperature reaches or surpasses 38.0℃, exercise training can significantly impair the recovery and function of the autonomic nervous system, with this effect intensifying as the temperature approaches 39.0℃ (26). This justifies the use of 38.5℃as a critical threshold, as physiological system dysfunction may have already commenced within this temperature range. Concurrently, the heart rate threshold is generally set at a sustained rate exceeding 150 beats per minute. This criterion reflects the excessive strain on the cardiovascular system under heat stress to accommodate the dual demands of heat dissipation and blood supply to the exercising muscles. A persistently elevated heart rate may signal that the compensatory mechanisms of the cardiovascular system have reached their limit, and the circulatory system can no longer effectively reconcile the conflict between skin blood flow for heat dissipation and muscle oxygenation, serving as a significant cardiovascular marker of heat intolerance (27). In the standard HTT judgment logic, these two criteria typically operate on an “or” relationship. This implies that during the test, as long as either the subject's core body temperature or heart rate surpasses its predetermined fixed threshold, they can be classified as “heat intolerant” (28). This binary judgment system is straightforward to implement and possesses clear benchmarks, aiming to swiftly identify individuals with aberrant physiological responses in hot environments and to mitigate health risks, providing a definitive basis for terminating the test and implementing intervention measures. To facilitate the interpretation of HTT results, Haggai Schermann et al. developed a logistic regression model using core body temperature and heart rate, and derived a new variable—the probability of heat tolerance (PHT)—to assess heat intolerance (29, 30). In this model, a PHT > 0.9 is considered heat tolerant, whereas a PHT < 0.5 is diagnosed as heat intolerance, indicating the need for heat acclimation training (Table 1).

**Table 1:** Current heat tolerance testing protocols.

HTT protocols	Ambient temperature, ralative humidity	Activity	Intensity	Equipment	Criteria for determining heat intolerance
Israeli (29, 30)	40℃, 40%	Walk	5 km/h Speed and 2% Incline	Shorts	Rectal temperature > 38.5℃, or heart rate > 150 bpm, or PHT < 0.5
United States (4)	40℃, 40%	Walk	5 km/h Speed and 2% Incline	Shorts	Rectal temperature > 38.5℃, or heart rate > 150 bpm
British (31)	34℃, 44%	Walk	60% of VO₂max	Military uniform plus a 15 kg backpack	Sustained rise in core temperature without a plateau
France (32, 33)	42℃, 20%	Three repetitions of an 8-minute run, with a 4-minute walk at 5 km/h between each running bout.	Running at 50% of VO₂max	Shorts	Rectal temperature > 38.5℃.

The British military protocol for heat intolerance assessment diverges markedly from the Israeli protocol in several key aspects, including the test environment, exercise load, attire, and criteria for judgment. Unlike the Israeli protocol, which is conducted in a controlled environment of 40℃ and 40% relative humidity, the British military protocol specifies a more temperate setting of 34℃ with a higher relative humidity of 44%. Participants are required to don full military attire and carry a 15 kg backpack during the initial phase of the test, which is designed to emulate the actual operational conditions faced by soldiers. The exercise intensity is calibrated to 60% of the individual's VO_2_max, reflecting a more strenuous physical demand. After 30 min of loaded walking, the subject can remove the backpack and jacket and continue walking for another 60–90 min. If the rectal temperature reaches a stable plateau, the patient is considered heat tolerant. Throughout the test, equipment is progressively removed in stages, which allows for a dynamic assessment of the subject's physiological response to changing conditions. The determination of heat tolerance is contingent upon whether the subject's rectal temperature plateaus (31).

The French protocol tends to conduct heat tolerance testing intermittently during heat acclimation training to assess the risk of heatstroke and the effectiveness of the training. Therefore, the French protocol also takes into account the feasibility of performing heat tolerance testing outdoors (32, 33). These variations underscore the significance of tailoring HTTs to the specific occupational or athletic profiles of the subjects, taking into account variables such as clothing, equipment, and load, to ensure a more accurate and relevant assessment of heat tolerance.

### 3.2 Key points for safety and quality control in HTT

Safe administration of HTT requires strict adherence to procedural guidelines and vigilant monitoring to ensure participant safety and control over testing quality. Standardized preparatory procedures are critical for obtaining reliable results. Participants should achieve optimal hydration before testing to avoid confounding effects of dehydration on core temperature and cardiovascular responses, which may compromise the accuracy of heat tolerance assessments (34). A brief warm-up routine helps participants acclimate to the elevated ambient temperature, ensuring reliable baseline physiological data. These quality control measures aim to minimize the influence of non-thermoregulatory variables on test outcomes.

Clear termination criteria are fundamental to both safety and quality control. In addition to established heat intolerance thresholds (e.g., rectal temperature > 38.5℃ or heart rate > 150 bpm), absolute safety limits are necessary. If core temperature reaches or exceeds 39.5℃, the test must be terminated immediately regardless of other metrics, serving as a critical failsafe against serious medical incidents such as exertional heatstroke. Moreover, any subjective symptoms indicating impending heat illness or extreme discomfort—including severe dizziness, nausea, vomiting, ataxia, or impaired coordination—should prompt immediate test cessation (34). Studies have shown that individuals with heat intolerance may exhibit significant declines in neurocognitive function and emotional stability during HTT even without reaching threshold physiological markers, highlighting the need for a comprehensive safety assessment that includes subjective symptoms (35). By rigorously implementing these safety and quality control measures, HTT can provide robust physiological evidence to support clinical decision-making within a controlled risk framework (36).

### 3.3 Limitations of HTT

HTT, while prevalent, faces notable limitations and debates. The use of static thresholds (e.g., rectal temperature at 38.5℃ heart rate at 150 bpm) fails to capture individual variability significantly influenced by factors such as age, fitness level, metabolic rate, body surface area-to-mass ratio, and cardiovascular health (27). Elite athletes or those with high physical fitness may tolerate heart rates above 150 bpm without impairment, yet rigid application of these standards could incorrectly label them as heat intolerant. Conversely, individuals with lower fitness levels or health conditions might experience physiological stress before reaching these thresholds.

The binary model of HTT, categorizing individuals as either “tolerant” or “intolerant,” is flawed as it does not gradate the degree of heat intolerance (25). This oversimplification hinders the development of tailored recovery and re-acclimatization plans. Moreover, HTT's reliance on core temperature and heart rate as primary indicators overlooks the nuanced physiological adaptations of heat acclimation, such as enhanced sweating rates, lower sweat onset thresholds, improved skin blood flow regulation, and plasma volume expansion (37). These adaptations are not assessed in standard HTT, potentially missing early signs of intolerance. Thus, future HTT must adopt a more personalized, quantitative, and multi-indicator approach to accurately assess heat tolerance and adaptation.

## 4 Heat tolerance reconstruction techniques

Heat tolerance training is a pivotal rehabilitative intervention aimed at facilitating the safe and efficient return to work for individuals who have experienced exertional heatstroke. The crux of this training lies in its ability to elicit a cascade of physiological and molecular adaptations through repeated exposure to thermal stress, thereby augmenting the body's heat tolerance (38). Studies have demonstrated that heat acclimition can significantly increase plasma volume, enhance cardiovascular stability, elevate sweating rates, and diminish core body temperature, heart rate, and perceived heat discomfort during physical exertion (39). This section primarily discusses the technical methods for heat tolerance reconstitution in patients with exertional heatstroke.

### 4.1 Heat tolerance training

**Figure 4:**
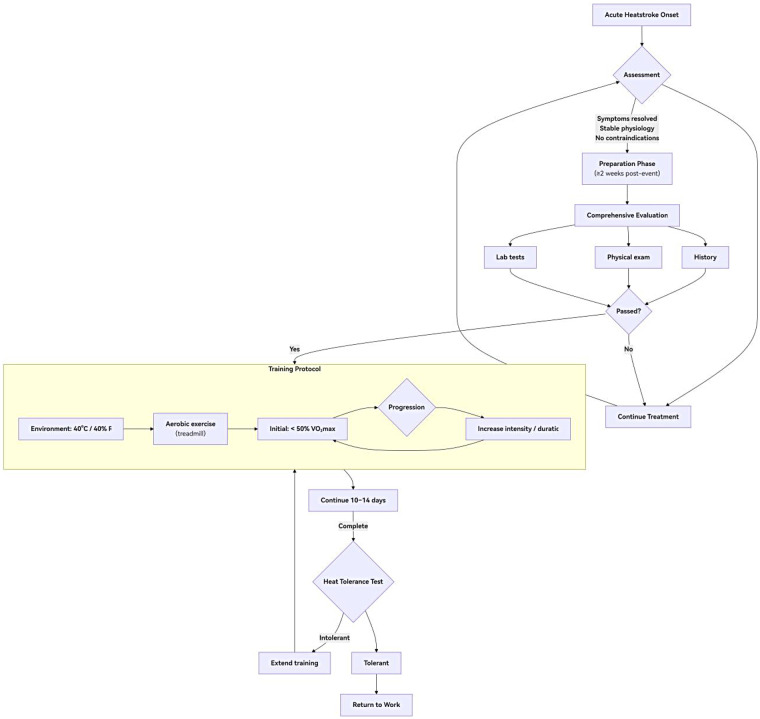
Heat tolerance training protocol for patients with heatstroke.

The initiation of heat tolerance training hinges on the patient's complete recovery from an acute heatstroke episode, ensuring the absence of ongoing multi-organ dysfunction (Figure 4). The precise timing for commencing training is largely contingent upon the dissipation of clinical symptoms and the stabilization of fundamental physiological parameters, given the absence of definitive biomarkers. This requires a thorough clinical evaluation, encompassing a meticulous review of medical history, physical examination, and requisite laboratory assessments to ascertain the absence of contraindications to training. While there is considerable variability in the onset timing, it is generally advised to initiate gradual heat tolerance training under stringent medical oversight no sooner than two weeks post-heatstroke event, provided all aforementioned safety criteria are satisfied (40). Premature initiation of training may exacerbate organ load or precipitate recurrent heatstroke, with potentially grave outcomes. Consequently, the formulation of a return-to-work plan must be predicated on the patient's specific clinical recovery profile.

The essence of heat tolerance training involves aerobic exercises conducted in a controlled thermal environment, designed to mimic actual workloads and elicit physiological adaptations. Common training regimens include treadmill walking or running and cycling (41). These activities are typically performed within an artificial climate chamber to ensure precise environmental control. Standard training conditions, as reported in literature, involve an ambient temperature of 40℃ and a relative humidity of 40%, effectively inducing the physiological stress necessary for heat acclimation. Training must adhere to the principle of progressive increments, with initial exercise intensity set at a low threshold, such as below 50% of the individual's VO2max, to mitigate heat stress risks and facilitate gradual adaptation. As training advances, the intensity, duration, or environmental heat stress can be incrementally escalated.

A comprehensive heat acclimation cycle typically spans 10 to 14 days, aligning with the classic timeframe for physiological heat adaptation. During this period, a daily heat exposure exercise session is standard, aiming to systematically lower core body temperature and heart rate, and enhance sweating rate and other pivotal physiological indicators through repetitive stimulation, thereby establishing stable heat tolerance. For general preventive heat adaptation in the population or athletes, this continuous two-week training cycle has proven effective in mitigating the risk of exertional heat illness (42). However, for individuals recovering from heatstroke, the total training duration often extends beyond, heavily contingent on the initial severity of heat intolerance and the individual's responsiveness to the training protocol. This underscores the significant individual variability in the rehabilitation training duration. Regarding training frequency, it is generally recommended to conduct 3 to 5 sessions per week (43), balancing the training stimulus with physical recovery to ensure adequate time for physiological adaptations to solidify while preventing increased risk of re-injury or impediments to recovery due to overtraining or cumulative fatigue.

### 4.2 Physical adjunctive therapies

Physical adjunctive therapies are integral to the rehabilitation process following exertional heatstroke, focusing on enhancing heat tolerance. These therapies include resistance training, transcutaneous heat therapy, and precooling strategies.

Resistance training is pivotal in the rehabilitation phase, aiming to bolster muscle strength and endurance. By improving muscular efficiency, it reduces metabolic heat generation during tasks, thereby mitigating the physiological strain of heat stress indirectly (44). Protocols should emphasize major muscle groups with moderate resistance and high repetitions to foster neuromuscular adaptation and muscle hypertrophy. This approach enhances muscle metabolic efficiency, decreasing heat production per muscle contraction and facilitating a lower relative effort level during work, which is vital for preventing recurrent heatstroke.

Transcutaneous heat therapy serves as a mild physical stimulus with potential benefits for vascular function and exercise tolerance, offering novel insights into cardiovascular recovery post-heat illness. Lower limb heat therapy has also been shown to reduce vasoconstrictor endothelin-1 levels and enhance physical perception (45). These findings suggest that transcutaneous heat therapy may augment recovery outcomes when used as a preparatory or adjunctive measure before heat acclimation training.

Precooling strategies are effective tools used to lower initial core and skin temperatures, thereby extending an individual's training tolerance in hot conditions (46). Techniques include ice vests, cold water immersion, and cold towels. Precooling reduces the body's “heat debt,” enabling patients to engage in more intense or prolonged heat exposure training, effectively triggering heat acclimation responses (47). In return-to-duty training, the strategic use of precooling can assist individuals in overcoming initial heat tolerance challenges, safely accumulating sufficient heat exposure, and fostering heat acclimation mechanisms, such as increased sweating and cardiovascular stability, ultimately aiming for unassisted tolerance of occupational heat loads.

### 4.3 Cognitive behavioral therapy

Cognitive restructuring is the initial phase, which aims to identify and modify patients’ exaggerated negative thoughts about heat stress and their physiological reactions. For example, patients may hold automatic negative thoughts such as, “I will collapse if I get hot again.” These thoughts can increase anxiety and lead to avoidance of heat, thereby hindering the recovery of heat tolerance and the return to work. Through structured exercises, Cognitive Behavioral Therapy（CBT） helps patients recognize the flaws in these thoughts and encourages them to adopt a more rational and constructive cognitive framework—for instance, understanding that “heat discomfort is a normal part of the body's reacclimatization process, and I can manage it effectively using learned strategies.” This cognitive change is essential for reducing fear-driven avoidance behaviors and establishing a psychological foundation for subsequent behavioral interventions.

Building on cognitive restructuring, CBT systematically and gradually help patients re-engage with and adapt to heat-related situations. The core of this approach is the development of a stepwise exposure plan, starting with low-risk situations and progressively increasing the level of challenge. The initial phase may consist of imaginal exposure, during which patients are guided to visualize themselves successfully coping with warm environments in a safe setting. Subsequently, they undergo real but controlled low-intensity heat exposure. The final phase integrates mild physical activity with supervised heat exposure training (48). This gradual exposure regimen operates on the principle of desensitization, reducing patients’ anxiety in response to heat stimuli and helping them regain psychological control over their physiological responses and the surrounding environment.

## 5 Outlook

The enduring heat intolerance observed in military personnel following exertional heatstroke represents a multifaceted pathophysiological phenomenon, characterized by regulatory imbalances across various systems and levels. This chronic state is fundamentally distinct from the acute phase of heatstroke, underscoring the necessity for tailored assessment frameworks and intervention strategies aimed at restoring heat tolerance post-exertional heat illness. Advancing the field requires a focus on three pivotal areas: Firstly, there is a critical need for an in-depth exploration of the mechanisms underpinning persistent heat intolerance. This includes elucidating the temporal and causal relationships among the diverse pathological components involved. Such insights are essential for developing targeted interventions. Secondly, the integration of cutting-edge technologies, such as wearable devices, multi-omics research, and biomarker identification, should be leveraged to refine rehabilitation protocols. The goal is to shift from a “one-size-fits-all” approach to one that is personalized and dynamically precise, thereby enhancing the efficacy of heat tolerance restoration. Lastly, there should be a concerted effort to conduct multicenter studies. These studies are vital for aggregating high-quality, evidence-based medical data. They will facilitate the continuous optimization of heat tolerance rebuilding processes and ultimately enhance the rate of successful return to duty for individuals who have suffered from heatstroke.
